# Inflammation associated with noise-induced hearing loss

**DOI:** 10.1121/1.5132545

**Published:** 2019-11-27

**Authors:** Mitchell D. Frye, Allen F. Ryan, Arwa Kurabi

**Affiliations:** 1 Callier Center for Communication Disorders, School of Behavioral and Brain Sciences, The University of Texas at Dallas, Dallas, Texas 75080, USA; 2 Department of Surgery/Otolaryngology, University of California San Diego, School of Medicine, and Veterans Administration Medical Center, La Jolla, California 92093, USA

## Abstract

Inflammation is a complex biological response to harmful stimuli including infection, tissue damage, and toxins. Thus, it is not surprising that cochlear damage by noise includes an inflammatory component. One mechanism by which inflammation is generated by tissue damage is the activation of damage-associated molecular patterns (DAMPs). Many of the cellular receptors for DAMPS, including Toll-like receptors, NOD-like receptors, and DNA receptors, are also receptors for pathogens, and function in the innate immune system. DAMP receptors are known to be expressed by cochlear cells, and binding of molecules released by damaged cells to these receptors result in the activation of cell stress pathways. This leads to the generation of pro-inflammatory cytokines and chemokines that recruit pro-inflammatory leukocytes. Extensive evidence indicates pro-inflammatory cytokines including TNF alpha and interleukin 1 beta, and chemokines including CCL2, are induced in the cochlea after noise exposure. The recruitment of macrophages into the cochlea has also been demonstrated. These provide substrates for noise damage to be enhanced by inflammation. Evidence is provided by the effectiveness of anti-inflammatory drugs in ameliorating noise-induced hearing loss. Involvement of inflammation provides a wide variety of additional anti-inflammatory and pro-resolution agents as potential pharmacological interventions in noise-induced hearing loss.

## I. INTRODUCTION

The inner ear was long treated as an immune privileged organ, implying that the tissues of the labyrinth are isolated from the systemic immune system and could tolerate the presence of external antigens without inducing an inflammatory response (McCabe, 1989). Part of this reasoning stemmed from early research that indicated the cochlear labyrinth lacks substantial lymphatic drainage (Harris and Ryan, 1984), although a few more recent studies have hinted that the labyrinth as a whole (including vestibular components) does in fact possess some degree of lymphatic drainage (Yimtae *et al.*, 2001). Additionally, the inner ear lies on one side of a tightly controlled blood-labyrinth barrier—extant in the stria vascularis—that separates the inner ear from general circulation (Harris and Ryan, 1984).

However, the tenets underlying cochlear immunoprivilege have been gradually reduced beginning with the work of Rask-Andersen and Stahle (1979), which revealed close interactions between lymphocytes and macrophages within labyrinthine tissues. Many recent studies have clearly demonstrated a robust cochlear immune capacity to noise stress. Transcriptome analyses of cochlear tissues have revealed that 80% of genes related to immune function are expressed in relatively constant amounts in cells of the cochlear sensory epithelium (Patel *et al.*, 2013; Cai *et al.*, 2014; Yang *et al.*, 2016). Subsequent to acoustic injury, many of these genes, which are related to immunity and inflammation, are up- or downregulated (Satoh *et al.*, 2002; Toubi *et al.*, 2004; Hirose *et al.*, 2005; Gazquez *et al.*, 2011; Yang *et al.*, 2015; Frye *et al.*, 2018), and many types of acoustic injury have been associated with an inflammatory cochlear response (Iwai *et al.*, 2003; Toubi *et al.*, 2004; Hirose *et al.*, 2005; Gazquez *et al.*, 2011; Yang *et al.*, 2015; Frye *et al.*, 2018). Quite promisingly, some of these inflammatory activities have been mitigated with the administration of anti-inflammatory drug treatments (Takahashi *et al.*, 1996; Sautter *et al.*, 2006; Fakhry *et al.*, 2007; Psillas *et al.*, 2008; Wakabayashi *et al.*, 2010; Zhou *et al.*, 2013). The importance of the inner ear's immune capacity in the event of noise exposure would seem evident. Several recent review articles on the subject of immunity and inflammation in the ear in response to stress and disease have been published (Goodall and Siddiq, 2015; Hirose *et al.*, 2017; Kalinec *et al.*, 2017; Wood and Zuo, 2017; Hu *et al.*, 2018). Interested readers may consult these references for additional information.

## II. INFLAMMATORY CELLS IN THE COCHLEA

It is well known that traumatic noise exposure results in cochlear damage and is particularly destructive to sensory cells (Taylor *et al.*, 1965; Sulkowski *et al.*, 1981; Bohne *et al.*, 2007). However, after substantial study of hair cell (HC) injury, researchers began to turn their attention to noise-induced damage in surrounding tissues and cells both in the sensory epithelium and in adjacent compartments (Wang *et al.*, 2002; Hirose and Liberman, 2003). Inflammation-associated cells were identified in noise-overexposed cochleae (Fredelius *et al.*, 1990; Fredelius and Rask-Andersen, 1990). Both sensory and supporting cells in the inner ear are prone to degeneration following noise insult, and though the organ of Corti itself is devoid of immune cells under resting conditions (Hirose *et al.*, 2005; Du *et al.*, 2011), surrounding labyrinthine tissues have been demonstrated to host immune cells derived from a hematopoietic cell line (Lang *et al.*, 2006; Okano *et al.*, 2008; Sato *et al.*, 2008).

### A. Inner ear macrophages

Numerous recent studies have shown that under both steady-state and pathological conditions, mature tissue macrophages are pervasive throughout major cochlear partitions including the stria vascularis, the spiral ligament, neural regions, and the basilar membrane (Lang *et al.*, 2006; Okano *et al.*, 2008; Sato *et al.*, 2008; Yang *et al.*, 2015). Moreover, infiltrated mononuclear phagocytes, including immature and less differentiated macrophages and monocytes, have been reported in many cochlear anatomic sites subsequent to cochlear stress. In ears that have undergone acoustic trauma, these infiltrated cells have been reported as present in the spiral ligament adjacent to fibrocytes (Hirose *et al.*, 2005; Tornabene *et al.*, 2006), in the scala vestibuli, modiolus, and lateral wall (Sautter *et al.*, 2006; Wakabayashi *et al.*, 2010; Du *et al.*, 2011), Reissner's membrane (Sautter *et al.*, 2006), and immediately beneath the basilar membrane in the scala tympani cavity (Frye *et al.*, 2017; Zhang *et al.*, 2017; Dong *et al.*, 2018; Frye *et al.*, 2018; Hu *et al.*, 2018).

Though the precise origin of these infiltrated monocytes is not entirely clear, it has been proposed these cells arrive from general circulation and enter cochlea via the blood-labyrinth barrier (Hirose *et al.*, 2005; Tornabene *et al.*, 2006; Okano *et al.*, 2008; Shi, 2010; Kaur *et al.*, 2015; Yang *et al.*, 2015). However, the presence of a substantial pool of small, undifferentiated leukocytes has been reported within the marrow of the bony labyrinth (Frye *et al.*, 2018). Thus, some or all of the infiltrated monocytes observed in the cochlea following stress may be derived from the bone tissue immediately surrounding the cochlea. Future studies aimed at elucidating the ultimate origin of these mononuclear phagocytes are needed.

Matern *et al.* (2017) report that fluorescence-activated cell sorting reveals 80% of the cochlea's immune cell composition is constituted by macrophages. Much of our current understanding of macrophage responses to cochlear stresses is derived from studies of acute acoustic injury, which culminate in the permanent loss of hearing sensitivity (Fredelius and Rask-Andersen, 1990; Hirose *et al.*, 2005; Tornabene *et al.*, 2006; Yang *et al.*, 2015). However, inflammatory activation of cochlear macrophages has also been demonstrably associated with low-level noise exposures that produce only temporary threshold shifts (Frye *et al.*, 2018).

Macrophages have been identified with the employment of immunohistochemistry in cochlear tissues. Numerous protein markers either specific to or strongly correlated with cochlear macrophages have been reported. These include the glycosylated transmembrane protein CD68 (Smith and Koch, 1987; Ramprasad *et al.*, 1996) and the mononuclear phagocytic marker F4/80 (Okano *et al.*, 2008), which is found to be strongly associated with cells of a highly-ramified morphology (Hume *et al.*, 2002). Ionized calcium-binding adapter molecule 1 (IBA1) is also reported to be macrophage-specific, and this calcium signal mediating protein has been purported to play an essential role in macrophage migration and phagocytosis (Imai *et al.*, 1996). Colony stimulating factor 1 receptor (CSF1R), also known as CD115, is found on the surface of macrophage membranes and is a specific cytokine receptor for the cytokine colony stimulating factor 1, which has been indicated in the regulation of mononuclear phagocyte survival and propagation (Hume *et al.*, 2002).

Cochlear macrophage distribution and responses to noise insult have been investigated by numerous researchers. In addition to the very presence of these cells being observed within the various cochlear partitions outlined above, an apical-to-basal gradient in macrophage phenotype has been observed in cochlear sensory epithelia under steady-state conditions (Yang *et al.*, 2015; Frye *et al.*, 2017). In particular, inner ear macrophages located immediately beneath the basilar membrane within the scala tympani cavity have received considerable attention, as these cells are the closest macrophages to sensory cells and the synapses for auditory spiral ganglia within the cochlea and are thus able to respond to stresses exerted on this tissue (Yang *et al.*, 2015; Frye *et al.*, 2017; Zhang *et al.*, 2017; Frye *et al.*, 2018; Hu *et al.*, 2018). While apically-located basilar membrane macrophages tend to display a ramified, dendritic morphology suggestive of resting and monitoring status, the basal turn of the basilar membrane presents with macrophages of an amoeboid morphology—a phenotype commonly seen in activated macrophages during noise-induced inflammation (Yang *et al.*, 2015).

This site-dependent morphology of basilar membrane macrophages points to an inborn immune capacity for cells of a dendritic shape versus amoeboid cells. While mature dendritic mononuclear phagocytes represent primarily latent immune cells engaged in monitoring the local tissue environment (Kreutzberg, 1996), cells of an amoeboid morphology epitomize a highly activated immune cell state associated with inflammation (Young and Bok, 1969; Vaughan and Peters, 1974; Peters and Swan, 1979; Kloss *et al.*, 1999; Stence *et al.*, 2001; Frye *et al.*, 2017; Frye *et al.*, 2018).

Major differences between macrophage responses provoked by acute and by chronic cochlear noise-induced pathogeneses have been demonstrated. In the event of acute noise overexposure, a large number of monocytes from circulation expeditiously infiltrate into the cochlea (Hirose *et al.*, 2005; Tornabene *et al.*, 2006; Okano *et al.*, 2008; Shi, 2010; Kaur *et al.*, 2015; Yang *et al.*, 2015). Therefore, in the event of traumatic and acute noise stresses, infiltrated macrophages are the major executor for inner ear immune activities, including phagocytosis of broken-down cellular material (Fredelius and Rask-Andersen, 1990) and the production of inflammatory molecules (Gloddek *et al.*, 2002; Fujioka *et al.*, 2006; Tornabene *et al.*, 2006; Yang *et al.*, 2015).

**FIG. 1.**
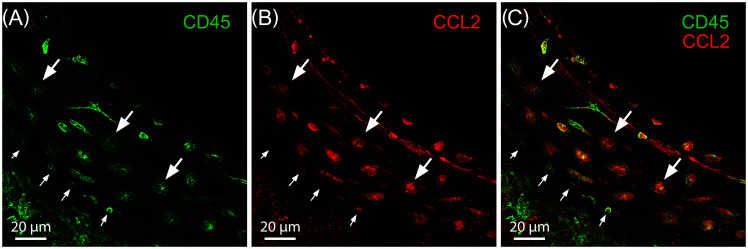
Inflammatory immune cells beneath the middle turn of the cochlear sensory epithelium four days following exposure to 120 dB sound pressure level (SPL) traumatic noise. (A) Pan-leukocyte marker CD45 expression showing myriad inflammatory mononuclear phagocytes. (B) CCL2 expression at the same anatomic site. (C) Note that CD45 and CCL2 expression is co-localized in both large mature amoeboid tissue macrophages (large arrows) and small recently infiltrated monocytes (small arrows). Scale bar = 20 *μ*m.

**FIG. 2.**
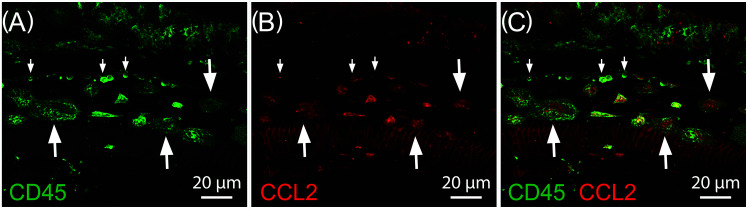
CD45 and CCL2 expression near the basal extreme of the cochlear sensory epithelium four days following exposure to 120 dB SPL traumatic noise. (A) CD45 allows for visualization of enlarged amoeboid macrophages (large arrows) and small less-differentiated mononuclear phagocytes along the lateral wall of the cochlea (small arrows). (B) CCL2 is expressed in both immune cells and non-immune supporting cells of the basal extreme following traumatic noise insult. (C) Merged image showing the co-localization of CD45 and CCL2 in the inflammatory cells of the cochlear basal extreme.

In contrast, during chronic lower level noise stress, the cochlear immune response is carried out primarily by mature resident tissue macrophages, though infiltration of proinflammatory monocytes expressing lymphocyte antigen 6 complex (Ly6C), C-C motif chemokine ligand 2 (CCL2) and the intercellular adhesion molecule 1 (ICAM-1) have also been reported (Frye *et al.*, 2018). Both of these mononuclear phagocyte subtypes display strong proinflammatory activation to noise-related sensory epithelial stress and degeneration, particularly in the portions of the sensory epithelium most susceptible to noise-induced insult including the middle turn of the basilar membrane as seen in Fig. 1, and the basal extreme as shown in Fig. 2 (Hirose *et al.*, 2005; Tornabene *et al.*, 2006; Yang *et al.*, 2015; Frye *et al.*, 2018). These findings highlight the importance of mature tissue macrophages and recently infiltrated monocytes in both the summative cochlear immune capacity and the inner ear's response to noise insult.

### B. Cochlear macrophages and microglia: Parallels in immune capacity and activation

In non-cochlear tissues, macrophages perform essential functions related to tissue homeostasis and pathogenesis. For example, microglia, the resident macrophages in the central nervous system (CNS), have been shown to play a complex role of either neuroprotection or destructive neuronal necrosis and apoptosis, depending on the degree of neurodegenerative insult (Banati *et al.*, 1993; Aschner *et al.*, 1999; Bruce-Keller, 1999; Murray and Wynn, 2011).

Many parallels can be drawn between microglia and mature tissue macrophages of the cochlea. Microglia are present even under steady-state conditions as several different phenotypes with individual morphologies believed to perform distinct immunological functions (Davis *et al.*, 1994; Raivich *et al.*, 1999). This same innate capacity to adopt specific phenotypes also appears immanent in the macrophages of the cochlea, particularly those macrophages along the sensory epithelia. Prior studies of sensory epithelium macrophages indicate a site-dependent morphology for apical versus basal portions of the basilar membrane, suggesting a manifest immune capacity for cells of a dendritic shape versus amoeboid cells (Yang *et al.*, 2015; Frye *et al.*, 2017; Zhang *et al.*, 2017) mirroring that observed for microglia in the CNS (Yin *et al.*, 2017).

Both microglia and mature tissue macrophages are responsible for the induction of the innate and adaptive immune response in the CNS (Davalos *et al.*, 2005; Nimmerjahn *et al.*, 2005) and the cochlea, (Yang *et al.*, 2015), respectively. Following insult, both (1) resident CNS microglia and infiltrated monocytes and (2) cochlear tissue macrophages along with cochlear infiltrated monocytes mount a complex defense against injury, and this includes the ingestion of dead cells and debris (Fredelius, 1988; Fredelius and Rask-Andersen, 1990; Davalos *et al.*, 2005; Nimmerjahn *et al.*, 2005; Hirose *et al.*, 2017) in addition to antigen presentation (Harris, 1984; Unanue, 1984; Steinman, 1991; Yang *et al.*, 2015). Moreover, both inner ear tissue macrophages and CNS microglia are present prenatally and subsequently develop into distinct, mature phenotypes within the local tissue environment (Varol *et al.*, 2015; Dong *et al.*, 2018) where they remain throughout adulthood (Frye *et al.*, 2017; Yin *et al.*, 2017).

Another interesting point of consideration is the substantial number of proinflammatory and anti-inflammatory cytokine receptors expressed in both CNS microglia and tissue macrophages including TNFα, IFNγ, IL-10, IL-1β, IFNα/β, and TGFβ (Galli *et al.*, 2011; Kalinec *et al.*, 2017). Additionally, resident macrophages of the nervous system possess glutamate receptors and have been demonstrated as being able to react to changes in the level of this neurotransmitter in the local tissue environment (Kreutzberg, 1996; Bruce-Keller, 1999). As previously discussed (see Sec. II A), the close proximity of the cochlear macrophages located immediately beneath the basilar membrane and their dynamic activation following noise stress (Hirose *et al.*, 2005; Yang *et al.*, 2015; Frye *et al.*, 2018) hints at the possibility that inner ear macrophages may also possess this capability, as glutamate is the major excitatory neurotransmitter in the auditory system and is found throughout the synapses located between sensory cells and the auditory spiral ganglia (Robbin and Thompson, 1978; Eybalin, 1993; Matsubara *et al.*, 1996; Kujawa and Liberman, 2009). Further, glutamate excitotoxicity within and around the organ of Corti has been reported in the event of acoustic trauma and is thought to contribute to the degeneration of sensory cells due to noise stress (Spoendlin, 1971; Liberman and Mulroy, 1982; Robertson, 1983). Determining whether cochlear macrophages are able to respond to a localized increase of neurotransmitters in the cochlear microenvironment, including but not limited to glutamate, is a particularly important avenue of future scientific investigation in understanding the precise relationship between inner HCs, their spiral ganglion innervations, and local cochlear macrophages following noise overexposure.

### C. Cochlear monocytes

Considerable and rapid monocyte infiltration into cochlear tissue occurs after traumatic noise stresses. This influx has been demonstrated to generally occur within approximately two to seven days following initial insult (Fredelius and Rask-Andersen, 1990; Hirose *et al.*, 2005; Tornabene *et al.*, 2006; Wakabayashi *et al.*, 2010). These monocytes have been positively identified within numerous labyrinthine partitions, but the greatest amassing of these immune cells has been confirmed in the spiral ligament and the scala tympani (Hirose *et al.*, 2005; Sautter *et al.*, 2006; Tornabene *et al.*, 2006; Miyao *et al.*, 2008; Du *et al.*, 2011). Once monocytes have penetrated cochlear tissues, they change their phenotype, mature into macrophages, and begin to adopt characteristics of inflammatory cells (Yang *et al.*, 2015). An exhaustive list of explicit roles performed by these mature phenotypes is still a matter of scientific inquiry.

### D. Perivascular melanocyte-like macrophages

Perivascular melanocyte-like macrophages (PVMs) are myeloid cells which express myriad macrophage protein markers: F4/80, CD68, CD11b, and major histocompatibility complex class II (MHCII) (Shi, 2010). These immune cells are extant in numerous bodily tissues such as the central nervous system and the retinal epithelium of the eye (Cuadros and Navascues, 1998; Hess *et al.*, 2004), and they are also found in close proximity to the vascular structures of the inner ear (Shi, 2010). As is the case for many myeloid-derived cells presenting the macrophage phenotype, PVMs are implicated in the immune defense to local noise-induced tissue insult and the subsequent repair of localized tissue. In the event of cochlear microenvironmental noise stress, both resident PVMs and newly recruited cells from general circulation participate in the immune response (Shi, 2009; 2010).

The stria vascularis is contained within the upper portion of the spiral ligament which in turn forms the outer wall of the cochlear duct. This cochlear partition has abundant small blood vessels and capillaries, and it is amongst these structures in which cochlear-associated PVMs can be found (Shi, 2009, 2010). Though melanocytes were first identified in proximity to the stria vascularis in the early 1990s (Matsunaga *et al.*, 1995), it took decades longer to determine the precise function these cells performed in the immune capacity of the inner ear. Within the past decade, these melanocyte-like cells have been found to present with protein markers (such as F4/80) closely associated with an inflammatory immune capability (Zhang *et al.*, 2012). A role for PVMs in the regulation of the blood-labyrinth barrier after noise trauma has been purported, as the specific depletion of PVMs in the region of the stria vascularis has been shown to be associated with weakened capillary structures which can no longer maintain the tightly-controlled barrier necessary to preserve a healthy balance of cochlear fluids and therefore normal cochlear homeostatic function (Hukee and Duvall, 1985; Shi, 2010; Zhang *et al.*, 2012).

### E. Lymphocytes in the inner ear

The precise distribution, activation, and immune role played by lymphocytes (B cells and T cells) in cochlear tissue is yet to be fully elucidated. Macrophages appear to be the major executor cell for immune capacity in the sensory epithelium and surrounding labyrinthine tissue following noise injury (Hirose *et al.*, 2005; Tornabene *et al.*, 2006; Yang *et al.*, 2015; Frye *et al.*, 2018). However, because macrophages have been identified upregulating expression of MHCII associated with antigen presentation after traumatic noise exposure (Yang *et al.*, 2015), important contributions to cochlear immune capacity are likely played by lymphocytes, as these leukocytes instigate adaptive immunity (Swain, 1983; Unanue, 1984; Grusby *et al.*, 1991; Steinman, 1991). Macrophages and lymphocytes such as B cells and T cells are found near the site of immune response in numerous tissues including the cochlea (Rask-Andersen and Stahle, 1979; Takahashi and Harris, 1988; Yang *et al.*, 2015; Matern *et al.*, 2017). Cell-cell interactions between macrophages and lymphocytes lead to antibody production. When macrophages engulf antigens from pathogens or damaged cells, those antigens are broken down into small pieces that are then displayed on the macrophage cell surface attached to special antigen-presenting molecules called MHC II (Swain, 1983; Grusby *et al.*, 1991). This same process occurs simultaneously on the surface of B cells. When T lymphocytes encounter antigen pieces on the macrophage and on B cells, this stimulates the B cells to turn on antibody production—an essential initiating component of an inflammatory response (Steinman, 1991). In conjunction with the antigen presentation function of cochlear macrophages, T cells and B cells are of vital importance in bridging the gap between innate immunity and adaptive immunity in higher order organisms (Swain, 1983; Unanue, 1984; Grusby *et al.*, 1991; Steinman, 1991). Under pathological stresses such as acoustic overstimulation, it is the basal turn of the sensory epithelium which suffers the most intense degree of trauma. Quite expectedly, it is also the basal region of the basilar membrane which sees the greatest degree of both monocyte infiltration, upregulation of antigen presentation, and an increase in the number of T cells (Takahashi and Harris, 1988; Gloddek *et al.*, 2002; Yang *et al.*, 2015).

Differential distribution, activation, and phenotype of hematopoietic-derived cells in cochlear tissue both under steady-state conditions and subsequent to noise-induced stress suggest that this heterogeneity is related to the inborn capacity of these immune cell populations to perform specialized functions within their respective cochlear microenvironments.

## III. GENE-REGULATED IMMUNE ACTIVATION IN NOISE-INDUCED COCHLEAR PATHOGENESIS

Several groups of researchers have investigated cochlear immunity by examining the molecular profiles of inner ear tissues under both steady-state and pathogenic conditions. For more than the past decade, investigators have applied techniques such as RNA sequencing—initially employed to analyze non-cochlear tissues (Beane *et al.*, 2011; Bottomly *et al.*, 2011; Huang *et al.*, 2011)—to gain a more comprehensive understanding of gene expression within tissues of the inner ear.

What seems clear from the literature is that a large number of immune-related genes are expressed in the cochlea under naive conditions (Cho *et al.*, 2004; Kirkegaard *et al.*, 2006; Tornabene *et al.*, 2006). Moreover, the upregulation of certain immune-related genes and the downregulation of others has been documented under numerous pathological conditions and subsequent to cochlear stresses (Cho *et al.*, 2004; Kirkegaard *et al.*, 2006; Tornabene *et al.*, 2006; Patel *et al.*, 2013; Cai *et al.*, 2014; Yang *et al.*, 2016).

### A. Adaptive levels of gene expression in the cochlea

The upregulation of myriad immune-related genes in the organ of Corti and surrounding inner ear tissues following cochlear insult, including noise overexposure, have been reported. These include, but are not limited to, TNF, CCL2, CCL4, and IL6 (Vethanayagam *et al.*, 2016), CXCL10, SOCS3, Ifrd1, Ifi202b, Igh-6 and TCl1b1 (Gratton *et al.*, 2011), CD68 and MHC II genes (Jabba *et al.*, 2006), CD45 and H2-Aa (Yang *et al.*, 2015), amongst many others. An exhaustive list of natively expressed immune-related genes in cochlear tissue is simply too extensive to list in its entirety here. However, differential immune-related gene expression levels in naive cochlear sensory epithelia (within the organ of Corti proper and in surrounding epithelial tissue) and in the same tissue subsequent to noise stress have been disseminated (Cai *et al.*, 2014; Yang *et al.*, 2016; Frye *et al.*, 2018). Additionally, genes related to inflammation have been described in the spiral ligament of the lateral wall (Fujioka *et al.*, 2014a) and within the stria vascularis (Jabba *et al.*, 2006). The presence and alterations in regulation of immunity genes in cochlear tissue following noise trauma are evident.

### B. Cellular signaling: Cytokines and chemokines

In addition to the upregulation and downregulation of particular immune-related genes, researchers have investigated myriad molecular pathways that are involved in the immune responses of inner ear tissues. Cytokine-cytokine receptor interaction, complement and coagulation cascades, chemokine signaling, NOD-like receptor (NLR) signaling (Yang *et al.*, 2016), and toll-like receptor signaling (Vethanayagam *et al.*, 2016; Yang *et al.*, 2016), amongst others, have all been reported to exist among cellular structures within cochlear tissues. Cytokines and chemokines are a broad category of cell signaling proteins. Their release sends specific signals from initiating signaling cells to surrounding cells in turn altering the behavior of cells in the local environment. They are implicated as potent immunemodulating agents. Of key importance is that cytokines and chemokines are produced by both immune cells such as macrophages, dendritic cells and microglia (Berti *et al.*, 2002; Arango Duque and Descoteaux, 2014) and non-immune cells alike. Understanding the intricate relationships associated with this cellular signaling provides promise in the direction of future therapeutic strategies aimed at modulating the immune response to noise trauma (Nishimoto and Kishimoto, 2006; Willrich *et al.*, 2015).

### C. The potential role of cytokine and chemokine signaling in hearing dysfunction

Increases in the presence of proinflammatory cytokines and chemokines have been reported in damaged cochleae (Vethanayagam *et al.*, 2016; Yang *et al.*, 2016). For example, the presence of TNF-α, IL-1β, IL-6, and MHCII have all been associated with monocyte infiltration during investigations into cochlear inflammation (Satoh *et al.*, 2002; 2003; Hashimoto *et al.*, 2005; Wakabayashi *et al.*, 2010; Yang *et al.*, 2015). The release of many cytokines and chemokines set off chain reactions, and the precise outcomes resultant of alteration in the presence of these cell signaling proteins is worthy of continued investigation. Future studies aimed at uncovering the intricate biological processes involved in the regulation of cytokines and chemokines may open the door for future targets of pharmacotherapy.

## IV. MECHANISMS OF INNER EAR INFLAMMATION FOLLOWING NOISE TRAUMA

### A. Pattern recognition receptors (PRRs) and damage-associated molecular patterns

The above evidence of inflammatory events occurring in the cochlea during noise-induced hearing loss (NIHL) suggests that intense noise exposure robustly activates inflammatory mechanisms, including the generation of inflammatory mediators and the recruitment and activation of immune/inflammatory cells. How this occurs is not as clear. However, there is ample evidence from other tissues that implicates the activation of innate immune receptors.

Noise damage to the cochlea occurs in a normally sterile space, and there is no evidence that infection is a consequence of acoustic overexposure. This indicates that any inflammation present in the inner ear after noise exposure must be the result of endogenous responses to cellular stress or damage. It has long been known that tissue damage without infection is inflammatory. However, only with the unraveling of innate immune PRRs and their ligands has a better understanding of the mechanisms involved emerged. Sterile inflammation in other organ systems has been strongly linked to the release of damage-associated molecular patterns (DAMPs) from stressed or damaged tissues (Tang *et al.*, 2012; Schaefer, 2014). DAMPs are recognized by many of the same innate immune PRRs that also respond to a wide variety of pathogen-associated molecular patterns (PAMPs). They contribute to the resolution of infection, and because they do not require prior sensitization, they are independent of cognate immunity (Takeuchi and Akira, 2010).

Why innate immune PRRs respond to DAMPs is not known. However, one possible reason is that many tissues, unlike the inner ear after noise, would be more susceptible to infection after damage, due to breaches in protective tissue barriers. Generating the innate immune effector cells and molecules that fight and resolve infections immediately after damage may give such tissues a head start in controlling infection. Alternatively, DAMP signaling may reflect the fact that inflammation can be beneficial to tissue healing and repair. In particular, macrophages have been found to have many functions in damaged tissue, including the promotion and resolution of inflammation, the removal of apoptotic or necrotic cells, support of cell proliferation and enhanced tissue restoration (Vannella and Wynn, 2017). Whatever the reason, it is clear that PRRs often play a significant role in tissue responses to damage.

### B. PRR Signaling

There are five general classes of PRRs: Toll-like receptors (TLRs), NLRs, retinoic acid-inducible gene 1 (RIG1)-like receptors, absent in melanoma 2 (AIM2)-like receptors, and C-type lectin receptors. There are several other molecules that can serve as PRRs, including POLIII, AIM, RAGE, and P2XR7. Just as these receptors collectively respond to a broad range of PAMPs, a property that underlies their ability to respond to a range of infections without prior sensitization, so too can they be activated by many DAMPS that are generated and released during tissue injury. Downstream from activated PRRs are pathways leading to the expression of many pro-inflammatory and anti-inflammatory mediators (Kumagai and Akira, 2010).

**FIG. 3.**
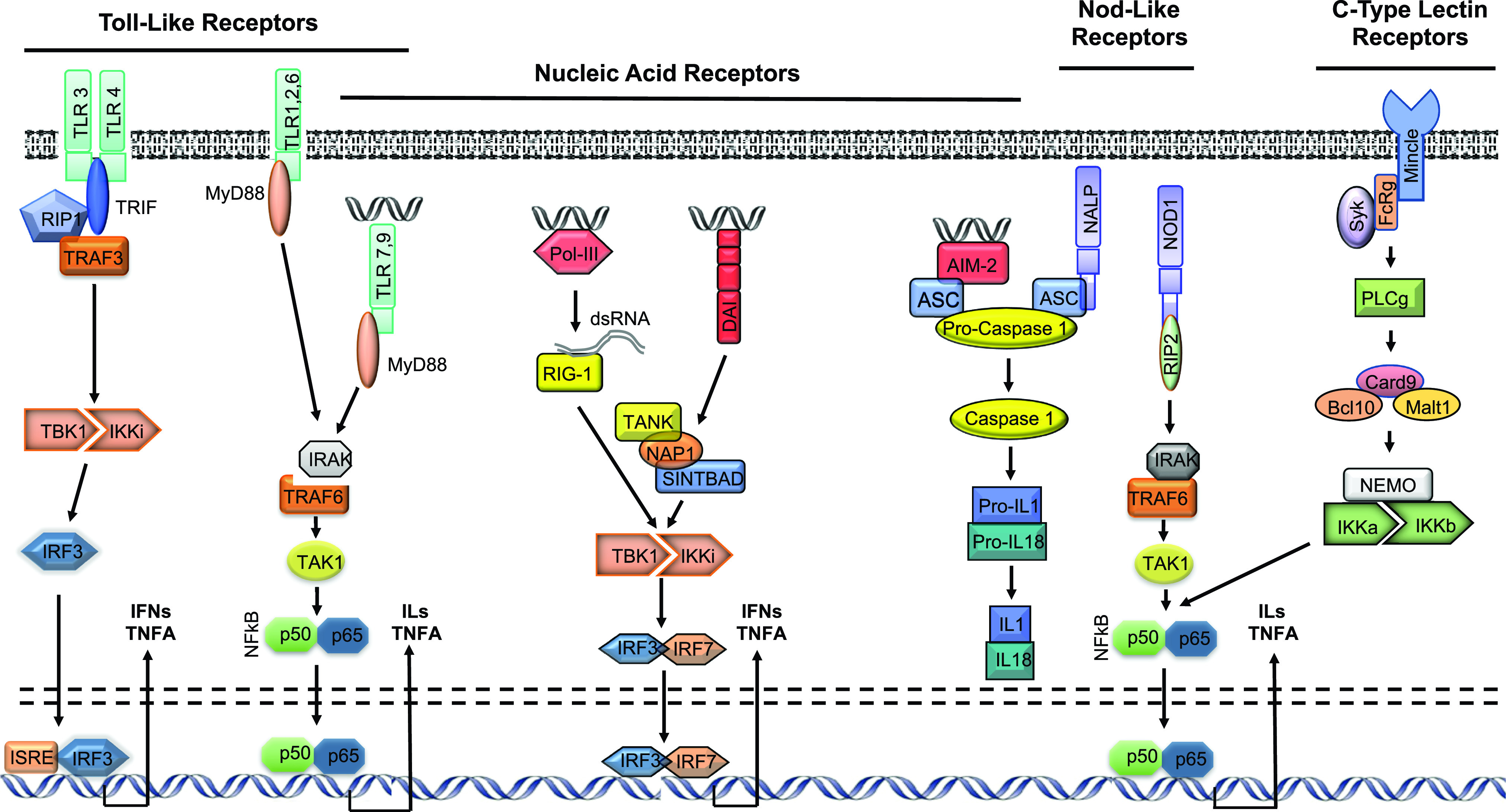
Schematic overview representation of the five major PRR families: TLRs, NLRs, RIG1-like receptors, AIM2-like receptors and C-type lectin receptors (adapted from Leichtle *et al.*, 2012 and Kurabi *et al.*, 2016).

Signaling by PRRs is diverse (Mogensen, 2009). The major PRR families and their signaling pathways are illustrated schematically in Fig. 3. The majority of TLRs signal via the MyD88 adaptor, activating NFκB or MAPK pathways to induce cytokine production. However, TLR3 signals via an alternative adaptor, TRIF, to activate IRF3 and interferon gene expression. TLR4 can signal via either adaptor. Most TLRs are transmembrane and surveil the extracellular environment. However, TLRs 7/8 and 9 are endosomal, reacting to DAMPs that are released from their normal cellular compartments or phagocytosed into endosomes. TLR3, activated by mRNA (Kariko *et al.*, 2004), can be either extracellular or endosomal. The NLRs are intracellular receptors. NOD1 and NOD2 signal via the RIP2 adaptor to activate NFκB or MAPK pathways, inducing cytokine production, while NLRPs combine with ASC and pro-caspase 1 to form an inflammasome, activating caspase 1, which in turn converts pro-IL1beta and pro-IL18 into their active forms. The RIG-1 like receptors, also intracellular, activate IRFs 3 and 7 and interferon production. C-type lectin receptors are transmembrane and detect extracellular lectins. They signal via PLC gamma to activate NFκB and MAPKs, leading to cytokine production.

The DAMPS that activate these receptors can be broadly divided into two categories: extracellular versus intracellular in origin (Schaefer, 2014). Extracellular DAMPs are primarily soluble, proteoglycan fragments of extracellular matrix molecules, generated by proteolysis or synthesized *de novo* by stimulated macrophages. Intracellular DAMPs include a wide variety of broken-down cellular materials and nuclear molecules that are released upon cell injury and death, including both DNA and RNA species (Roers *et al.*, 2016). A list of DAMPs recognized by various PRRs is presented in Table I.

**TABLE I.** DAMP recognition by innate immune receptors.

Receptor	DAMPs
*Toll-like Receptors*	
TLR1	Beta-defensin3
TLR2	HSP60, 70, 90
	HMGB1
	Histones
	Surfactant protein A, D
	Eosinophil-derived neurotoxin
	Anti-phospholipid antibodies
	ECM-derived/secreted: biglycan, decorin, versican, LMW hyaluronan
	S100 proteins
RAGE	amyloid beta
	HMGB1
	S100 proteins
	dsDNA fragments
	dsRNA fragments
TLR3	mRNA
TLR4	HSP60,70
	HMGB1
	Histones
	ECM-derived/secreted: biglycan, decorin, LMW hyaluronan, heparan
	sulfate, fibronectin EDA isoforms, tenascin C, fibrinogen
	S100 proteins
	Membrane-derived: syndicans, glypigans
TLR6	Versicans
TLR7	dsRNA fragments
	Cathelicidin
TLR8	dsDNA fragments
TLR9	mtDNA
	dsDNA fragments
	Cathelicidin
	HMGB1
*NOD-like receptors*	
NLRP3	Uric acid
	Mitochondrial ROS
	Hyaluronan
	Histones
*RIG-1-like receptors*	
RIG-1	dsRNA
MDA5	dsRNA
*AIM2-like Receptors*	
AIM2	dsDNA fragments
MNDA	dsDNA fragments
PYHIN1dsDNA fragments	
IFI16	dsDNA fragments
*C-type Lectin Receptors*	
Mincle	SAP130
*Additional DAMP receptors*	
POLIII	dsDNA
cGAS	dsDNA
DAI	dsDNA
P2XR7	ATP

### C. PRRs and DAMPs in cochlear cells

Although PRRs are widely expressed by many cell types, it is less clear which of those listed in Table I might be present in cochlear cells. While gene expression databases for many cochlear cell types are not readily available, those for HCs are. We therefore examined the Shield database of mRNAs that are expressed by normal adult mouse inner and outer HCs, as determined using gene arrays (Liu *et al.*, 2014). Positive hybridization to probes for all of the PRRs listed in Table I was observed for both inner and outer HCs. While gene array hybridization levels are not directly related to expression levels, levels were modest for most PRRs, while the levels for *Cgas* (E330016A19Rik), *Pylin1*, *Ifi16* (*Ifi204* in mouse), and *Clec4e* (Mincle) were very low.

It is obvious that damaged HCs and other cochlear tissues could release universal cell constituents such as nucleic acids, histones, membrane-derived molecules and mitochondrial reactive oxygen species (ROS). Many other DAMPs can be common cell constituents. However, we employed the same expression database as above to assess their presence in HCs. Most of the DAMPs listed in Table I were expressed by HCs, although as expected the genes for cathelicidin, eosinophil-derived neurotoxin and antibodies were absent. Particularly high levels of hybridization were noted for probes against Hsp60, Hsp90, S100a6, and S100a13 mRNAs. Finally, expression of most of the pathway genes linking PRRs to the nucleus (Fig. 3) was observed. These gene expression data suggest that the substrates for DAMP signaling are present in the cells most affected in NIHL, even before acoustic trauma. Damaged inner ear cells that maintain some degree of integrity could expose their intracellular PRRs to PAMPs, including many TLRs and NLRs as well as various nucleic acid receptors. Necrotized cells would be expected to release significant amounts of DAMPs into the extracellular environment, activating transmembrane PRRs such as many TLRs and C-type lectin receptors. Cells undergoing apoptosis would similarly expose the intracellular PRRs of phagocytes to DAMPs.

Activation of PRRs by DAMPs results in the expression of a large number of molecules, including pro- and anti-inflammatory cytokines, chemokines that recruit various classes of leukocytes, host defense molecules, immunomodulators and opsonization markers, as well as growth factors that stimulate tissue growth and healing (Tran *et al.*, 2012; Schaefer, 2014). Of particular importance to innate immune responses in sterile tissue damage are macrophages. Macrophages express a very wide variety of innate immune molecules (Gasteiger *et al.*, 2017). Resident macrophages in tissue perform important surveillance for PAMPs and DAMPs and are recruited to sites of damage from the circulation. As we have seen above, resident cochlear macrophages are clearly involved in NIHL. While classically activated (M1) macrophages observed early in inflammation produce many pro-inflammatory mediators, tissue recovery is enhanced by the action of the several alternatively activated (M2) phenotypes that are found after the initial burst of inflammation is complete, in response to the secretion of anti-inflammatory cytokines including IL-10 and IL-4. The primary role of M2 macrophage in tissue injury is the phagocytosis of damaged tissue. The macrophages also release proteases that can debride damaged tissue and enhance phagocytic activity. During later stages of injury they secrete growth factors that can enhance cell survival, stimulate cells involved in healing and generate new extracellular matrix.

### D. Assessing the role of PRRs in NIHL

Given the wide variety of PRRs and their overlapping ligand specificity, it can be difficult to assign a role in a disease or damage process to specific PRRs. One possible avenue to address this problem would be to study NIHL in gene deletion mice (e.g., TLR4 knockout; Vethanayagam *et al.*, 2016). Many such experiments have been performed in other systems to evaluate the role of various individual PRRs in disease models. A case in point is otitis media, where it has been found that while many PRRs contribute to infection resolution, a few play a more significant role in regulation of inflammatory responses (Kurabi *et al.*, 2016). Use of mice lacking obligatory signaling molecules that target more than one PRR, such as MyD88 or RIP2, could facilitate such investigations.

Another potential approach is PRR inhibitors. A number of PRR inhibitors with varying degrees of specificity have been developed. For example, TLR4 inhibitors that block its interaction with MD20 or with downstream signaling pathways have been developed (Kuzmich *et al.*, 2017). It has also been reported that some phytochemicals block TLR4 signaling by preventing receptor dimerization (Zhao *et al.*, 2011). Inhibitors of TLR2 (Mistry *et al.*, 2015), of TLR3 interaction with mRNA (Takemura *et al.*, 2014), NLRP3 (Perera *et al.*, 2018) and RAGE (Bongarzone *et al.*, 2017) have also been developed. Evaluating a panel of such inhibitors might identify critical components of DAMP signaling in NIHL. This approach also has the advantage of identifying potential treatments that could ameliorate the effects of noise damage or enhance recovery.

## V. IMMUNE-MEDIATED PATHOGENESIS IN THE INNER EAR: POTENTIAL TREATMENTS, THERAPEUTIC TARGETS, AND INTERVENTIONS

Cochlear immunity is an important component of inner ear homeostasis, and alterations in a provoked immune response following cochlear stresses has been implicated in a number of inner ear pathogeneses. Several interventions and attempted mediations of the cochlear immune response have been investigated by numerous researchers. Many novel pharmacological agents, chemical signaling proteins, and pre- and post-conditioning paradigms have been suggested as treatments for immune-mediated cochlear disease.

### A. Sensorineural hearing loss (SNHL) and immunologically-induced cochlear pathogeneses

SNHL is a common clinical condition resulting from the dysfunction in one or more parts of the auditory pathway between the inner ear and the auditory cortex of the central nervous system. Inflammation initiation and resolution is essential for tissue homeostasis in many bodily tissues, and this includes tissues of the membranous labyrinth. In fact, an immune response has now been linked to or associated with all major causes of acquired hearing loss (Iwai *et al.*, 2003; Toubi *et al.*, 2004; Oh *et al.*, 2012; Fujioka *et al.*, 2014b; Goodall and Siddiq, 2015; Tan *et al.*, 2016).

A frequent factor in the induction of SNHL is excessive noise exposure. In fact, Moscicki *et al.* (1994) suggest that a minimum of 30 dB of bilateral SNHL with measurably decreasing thresholds in either one or both ears at two consecutive audiometric evaluations within three months could be used as a clinical guideline to suspect an underlying immunological constituency. Myriad inner ear diseases have been suggested to be due at least in part to immune system activation in the cochlea. For example, researchers have reported that patients with Meniere's disease demonstrate substantial recovery from symptoms such as fluctuating SNHL, vertigo, and roaring tinnitus after receiving systemic corticosteroid treatment, and the evidence points to an underlying immune-mediated component to this disease (Hughes *et al.*, 1983; Derebery *et al.*, 1991). Some multisystem autoimmune diseases including Wegener's granulomatosis, Cogan syndrome, Lupus (McCabe, 1989; Moscicki *et al.*, 1994), and even Crohn's disease (Dettmer *et al.*, 2011) have been implicated in cochlear pathogeneses. Though speculative, the strong immune activity associated with these multisystem autoimmune diseases may be responsible for some degree of cochlear tissue degeneration leading to SNHL secondary to the systemic autoimmune disorder. Moreover, limited studies suggest acute extreme inflammation within cochlear soft tissues occurs in patients presenting with sudden deafness or other SNHL of unknown origin as revealed by magnetic resonance imaging (Stokroos *et al.*, 1998). This inner ear inflammation has been demonstrated to at least partially subside if not completely resolve in patients who experience recovery from instances of transient idiopathic SNHL (Mark *et al.*, 1992). These studies make a case for an immunological component to many diseases affecting the inner ear.

In light of this, and due to the strong cochlear immune activity observed following acoustic stress (Fredelius and Rask-Andersen, 1990; Hirose *et al.*, 2005; Tornabene *et al.*, 2006; Wakabayashi *et al.*, 2010; Yang *et al.*, 2015; Frye *et al.*, 2018), there is reason to suspect the cochlear immune response may also play a role in the progression and final outcome of NIHL. Previous studies have demonstrated a time-dependent activation and subsidence of the cochlear immune response, including tissue macrophage activity in addition to monocyte infiltration beginning 2–7 days after acute noise insult (Fredelius and Rask-Andersen, 1990; Hirose *et al.*, 2005; Tornabene *et al.*, 2006; Wakabayashi *et al.*, 2010; Yang *et al.*, 2015). This immune activity is associated with substantial sensory cell degeneration and permanent hearing loss (Yang *et al.*, 2015). Further, cochlear immune capacity is also temporally associated with temporary threshold shifts caused by chronic lower-level noise stress (Frye *et al.*, 2018). Future studies are certainly needed to determine if off-target effects of robust cochlear immune activity contribute to the degree of SNHL following noise-induced injury.

### B. Immune cells of the inner ear as a specific therapeutic target

Macrophages appear to hold the primary immune capacity within cochlear tissues. These particular myeloid-derived cells differentiate from less differentiated precursor cells called monocytes. Both mature, resident tissue macrophages and newly-arrived monocytes have been reported in numerous cochlear partitions under naive conditions and subsequent to noise exposure (Hirose *et al.*, 2005; Tornabene *et al.*, 2006; Okano *et al.*, 2008; Sato *et al.*, 2008; Yang *et al.*, 2015). Due to the involvement of these immune cells during the time course of both acute (Fredelius and Rask-Andersen, 1990; Hirose *et al.*, 2005; Tornabene *et al.*, 2006; Yang *et al.*, 2015) and chronic noise stresses (Frye *et al.*, 2018), it seems that macrophages and monocytes could be targeted in attempts to mediate immunologically influenced cochlear pathogeneses caused by excessive noise exposure.

Macrophages can present as either M1-proinflammatory or M2-anti-inflammatory cells. However these two phenotypes in fact only represent two extremes on a continuum of macrophage functional capacity (Lawrence and Natoli, 2011). The regulation of cells exhibiting either of these two phenotypes is controlled by microenvironmental signaling including variable concentrations of particular chemokines and cytokines. Under typical immune response conditions, following a typical time course of immune activation and subsidence, proinflammatory M1 macrophages either undergo cell death by means of apoptosis or alter their phenotype and transition to M2-anti-inflammatory macrophages (Lawrence and Natoli, 2011). Provided this tightly-controlled system is maintained, the immune response is essential in preserving local tissue homeostasis. However, if this pro-inflammatory/anti-inflammatory system becomes dysregulated, such as in the event of noise injury, the inflammatory response may become detrimental to the survival of cells in the local environment. Many factors, not all of which are either fully understood or have been described in their entirety, have been implicated in the dysregulation of the pro-/anti-inflammatory immune activity in numerous bodily tissues. Though current evidence for the dysregulation of pro-/anti-inflammatory effects specifically within cochlear tissue is lacking, the authors speculate that future investigations into the complex process of proinflammatory and anti-inflammatory activity of cochlear immune cells following noise trauma may illuminate new avenues for immunomodulation and hearing preservation.

### C. Steroid therapy as a potential clinical tool to control the inflammatory response

Treatments of SNHL have now long included the administration of corticosteroids. Both systemic treatment and local application of these pharmacotherapies have been utilized (Spear and Schwartz, 2011), but details regarding precisely the correct dosage, route of administration and time course of treatment are yet to be elucidated. Though the efficacy of corticosteroid treatment has been established, the therapeutic response is sometimes only short-lived (Zeitoun *et al.*, 2005), and many negative side effects of steroid treatment have been reported (Alexander *et al.*, 2009).

Due to myriad side effects associated with current corticosteroid therapy, the search is on for other possible immune-regulating pharmacotherapies (Harris *et al.*, 2003; Garcia-Berrocal *et al.*, 2006). Systemic immunosuppressive medications traditionally used to prevent tissue rejection following organ transplantation such as azathioprine (Lasak *et al.*, 2001) and cancer chemotherapeutics such as methotrexate (Matteson *et al.*, 2001; Salley *et al.*, 2001) have both been explored as alternatives to traditional corticosteroid treatments for immune-related NIHL. However, the results of these trials are less than conclusive in demonstrating efficacy (Harris *et al.*, 2003). Clearly, a search for further alternatives to steroid therapy for NIHL should continue.

### D. Biopharmaceuticals and proposed future treatments of immune-mediated SNHL

Numerous recent medical advances have provided for a promising outlook on immunotherapy to regulate inflammatory responses in the inner ear following noise injury. Molecular-specific targets have been of particular interest to basic scientists and clinicians alike. One of these primary targets is the tumor necrosis factor (TNF) superfamily, a large group of inflammatory cytokines responsible for the acute phase of tissue inflammation. Pharmacotherapies such as etanercept (Enbrel^®^), a drug designed to treat autoimmune disease by altering the functionality of TNF (Mohler *et al.*, 1993), has been evaluated in the treatment of immune-mediated inner ear disorders. In select patients with immune-mediated cochleovestibular disorders, Rahman *et al.* (2001) purport some limited efficacy of this biopharmaceutical in (1) preventing worsening of hearing loss as determined by improvement or stabilization of air conduction pure-tone audiograms and speech discrimination and (2) in the reduction of tinnitus symptoms as determined by a qualitative three-level response scale of better, no change, or worse. However, this retrospective, pilot study was performed with only 12 patients suspected of having immune-mediated inner ear disorders. Moreover, it lacked an appropriate control group. The benefit achieved by the administration of this molecule-specific therapy in the improvement of hearing function requires prospective studies with a larger sample size and appropriate experimental controls (Cohen *et al.*, 2005; Matteson *et al.*, 2005).

Other biopharmaceuticals have also recently been evaluated for treatment of immune-mediated inner ear disease. Both infliximab (Remicade^®^) and adalimumab (Humira^®^)—two additional TNF disruptors (Siddiqui and Scott, 2005; Morovic Vergles *et al.*, 2010)—have been investigated. Though Liu *et al.* (2011) reported no hearing improvement with the administration of infliximab in patients with immune-mediated SNHL, Van Wijk *et al.* (2006) and fellow researchers describe improvement in hearing thresholds and reduced recurring symptoms in a small cohort of nine patients who suffered from inner ear disease who were administered this drug transtympanically.

Another biopharmaceutical of interest is rituximab (Rituxan^®^), which is a medication employed in the treatment of autoimmune diseases such as rheumatoid arthritis and certain types of cancer such as non-Hodgkin's lymphoma. This molecular-specific agent targets CD20, a cell membrane surface protein located on the surface of B cells and affects the ability of these lymphocytes to produce antibodies targeting host tissues (Cohen *et al.*, 2011). A pilot study investigating rituximab in the treatment of patients with immunity-related hearing loss was conducted by Cohen *et al.* (2011), but this study suffers from poor design and lacked a randomized control group. Additional evaluation of this biopharmaceutical seems warranted.

Though numerous therapeutic agents such as corticosteroids and biopharmaceuticals have been investigated, in the future, even more aggressive and specific therapies may be available to treat immune-mediated noise-induced inner ear disorders such as pluripotent stem cell transplantation into the inner ear (Hakuba *et al.*, 2005; Okano *et al.*, 2006) and gene therapy targeting cochlear mononuclear phagocytes (Kesser and Lalwani, 2009).

## VI. CONCLUDING REMARKS

Mammals are able to detect pathogens and elements of tissue injury through multiple innate immune receptors that in turn initiate tissue repair and healing. The cochlear response to a noise trauma paradigm is based on the premise of inflammation that results from the engagement and activation of specific genetic, cellular, and molecular pathways that are perturbed within the micromachinery of the inner ear. The initiation and resolution of inflammation in the inner ear seem highly likely to involve innate immune responses to the release of endogenous molecules both within and outside of cochlear cells. PRRs and DAMPs are present in the cochlea and in HCs, and many genes that are activation targets of innate immune signaling pathways are expressed following acoustic trauma. Research to unravel the complexities of innate immune participation in NIHL would be productive to increase our understanding and reveal possible therapeutic solutions to acoustic injury.

## References

[1] Alexander, T. H. , Weisman, M. H. , Derebery, J. M. , Espeland, M. A. , Gantz, B. J. , Gulya, A. J. , Hammerschlag, P. E. , Hannley, M. , Hughes, G. B. , Moscicki, R. , Nelson, R. A. , Niparko, J. K. , Rauch, S. D. , Telian, S. A. , Brookhouser, P. E. , and Harris, J. P. (2009). “ Safety of high-dose corticosteroids for the treatment of autoimmune inner ear disease,” Otol. Neurotol. 30, 443–448. 10.1097/MAO.0b013e3181a52773 19395984

[2] Arango Duque, G. , and Descoteaux, A. (2014). “ Macrophage cytokines: Involvement in immunity and infectious diseases,” Front. Immunol. 5, 491 10.3389/fimmu.2014.00491 25339958 PMC4188125

[3] Aschner, M. , Allen, J. W. , Kimelberg, H. K. , LoPachin, R. M. , and Streit, W. J. (1999). “ Glial cells in neurotoxicity development,” Ann. Rev. Pharmacol. Toxicol. 39, 151–173. 10.1146/annurev.pharmtox.39.1.151 10331080

[4] Banati, R. B. , Gehrmann, J. , Schubert, P. , and Kreutzberg, G. W. (1993). “ Cytotoxicity of microglia,” Glia 7, 111–118. 10.1002/glia.440070117 8423058

[5] Beane, J. , Vick, J. , Schembri, F. , Anderlind, C. , Gower, A. , Campbell, J. , Luo, L. , Zhang, X. H. , Xiao, J. , Alekseyev, Y. O. , Wang, S. , Levy, S. , Massion, P. P. , Lenburg, M. , and Spira, A. (2011). “ Characterizing the impact of smoking and lung cancer on the airway transcriptome using RNA-Seq,” Cancer Prev. Res. (Phila) 4, 803–817. 10.1158/1940-6207.CAPR-11-0212 21636547 PMC3694393

[6] Berti, R. , Williams, A. J. , Moffett, J. R. , Hale, S. L. , Velarde, L. C. , Elliott, P. J. , Yao, C. , Dave, J. R. , and Tortella, F. C. (2002). “ Quantitative real-time RT-PCR analysis of inflammatory gene expression associated with ischemia-reperfusion brain injury,” J. Cereb. Blood Flow Metab. 22, 1068–1079. 10.1097/00004647-200209000-00004 12218412

[7] Bohne, B. A. , Harding, G. W. , and Lee, S. C. (2007). “ Death pathways in noise-damaged outer hair cells,” Hear Res. 223, 61–70. 10.1016/j.heares.2006.10.004 17141990

[8] Bongarzone, S. , Savickas, V. , Luzi, F. , and Gee, A. D. (2017). “ Targeting the receptor for advanced glycation endproducts (RAGE): A medicinal chemistry perspective,” J. Med. Chem. 60, 7213–7232. 10.1021/acs.jmedchem.7b00058 28482155 PMC5601361

[9] Bottomly, D. , Walter, N. A. , Hunter, J. E. , Darakjian, P. , Kawane, S. , Buck, K. J. , Searles, R. P. , Mooney, M. , McWeeney, S. K. , and Hitzemann, R. (2011). “ Evaluating gene expression in C57BL/6J and DBA/2J mouse striatum using RNA-Seq and microarrays,” PLoS One 6, e17820 10.1371/journal.pone.0017820 21455293 PMC3063777

[10] Bruce-Keller, A. J. (1999). “ Microglial-neuronal interactions in synaptic damage and recovery,” J. Neurosci. Res. 58, 191–201. 10.1002/(SICI)1097-4547(19991001)58:1<191::AID-JNR17>3.0.CO;2-E 10491582

[11] Cai, Q. , Vethanayagam, R. R. , Yang, S. , Bard, J. , Jamison, J. , Cartwright, D. , Dong, Y. , and Hu, B. H. (2014). “ Molecular profile of cochlear immunity in the resident cells of the organ of Corti,” J. Neuroinflamm. 11, 173 10.1186/s12974-014-0173-8 PMC4198756 25311735

[12] Cho, Y. , Gong, T. W. , Kanicki, A. , Altschuler, R. A. , and Lomax, M. I. (2004). “ Noise overstimulation induces immediate early genes in the rat cochlea,” Brain Res. Mol. Brain Res. 130, 134–148. 10.1016/j.molbrainres.2004.07.017 15519684

[13] Cohen, S. , Roland, P. , Shoup, A. , Lowenstein, M. , Silverstein, H. , Kavanaugh, A. , and Harris, J. (2011). “ A pilot study of rituximab in immune-mediated inner ear disease,” Audiol. Neurootol. 16, 214–221. 10.1159/000320606 20980741

[14] Cohen, S. , Shoup, A. , Weisman, M. H. , and Harris, J. (2005). “ Etanercept treatment for autoimmune inner ear disease: Results of a pilot placebo-controlled study,” Otol. Neurotol. 26, 903–907. 10.1097/01.mao.0000185082.28598.87 16151336

[15] Cuadros, M. A. , and Navascues, J. (1998). “ The origin and differentiation of microglial cells during development,” Prog. Neurobiol. 56, 173–189. 10.1016/S0301-0082(98)00035-5 9760700

[16] Davalos, D. , Grutzendler, J. , Yang, G. , Kim, J. V. , Zuo, Y. , Jung, S. , Littman, D. R. , Dustin, M. L. , and Gan, W. B. (2005). “ ATP mediates rapid microglial response to local brain injury in vivo,” Nat. Neurosci. 8, 752–758. 10.1038/nn1472 15895084

[17] Davis, E. J. , Foster, T. D. , and Thomas, W. E. (1994). “ Cellular forms and functions of brain microglia,” Brain Res. Bull. 34, 73–78. 10.1016/0361-9230(94)90189-9 8193937

[18] Derebery, M. J. , Rao, V. S. , Siglock, T. J. , Linthicum, F. H. , and Nelson, R. A. (1991). “ Meniere's disease: An immune complex-mediated illness?,” Laryngoscope 101, 225–229. 10.1288/00005537-199103000-00001 1825685

[19] Dettmer, M. , Hegemann, I. , and Hegemann, S. C. (2011). “ Extraintestinal Crohn's disease mimicking autoimmune inner ear disease: A histopathological approach,” Audiol. Neurootol. 16, 36–40. 10.1159/000315063 20523038

[20] Dong, Y. , Zhang, C. , Frye, M. , Yang, W. , Ding, D. , Sharma, A. , Guo, W. , and Hu, B. H. (2018). “ Differential fates of tissue macrophages in the cochlea during postnatal development,” Hear Res. 365, 110–126. 10.1016/j.heares.2018.05.010 29804721 PMC6026078

[21] Du, X. , Choi, C. H. , Chen, K. , Cheng, W. , Floyd, R. A. , and Kopke, R. D. (2011). “ Reduced formation of oxidative stress biomarkers and migration of mononuclear phagocytes in the cochleae of chinchilla after antioxidant treatment in acute acoustic trauma,” Int. J. Otolaryngol. 2011, 612690 10.1155/2011/612690 21961007 PMC3179894

[22] Eybalin, M. (1993). “ Neurotransmitters and neuromodulators of the mammalian cochlea,” Physiol. Rev. 73, 309–373. 10.1152/physrev.1993.73.2.309 8097330

[23] Fakhry, N. , Rostain, J. C. , and Cazals, Y. (2007). “ Hyperbaric oxygenation with corticoid in experimental acoustic trauma,” Hear Res. 230, 88–92. 10.1016/j.heares.2007.05.005 17590548

[24] Fredelius, L. (1988). “ Time sequence of degeneration pattern of the organ of Corti after acoustic overstimulation. A transmission electron microscopy study,” Acta Otolaryngol. 106, 373–385. 10.3109/00016488809122260 3207005

[25] Fredelius, L. , Johansson, B. , Bagger-Sjoback, D. , and Wersall, J. (1990). “ Time-related changes in the guinea pig cochlea after acoustic overstimulation,” Ann. Otol. Rhinol. Laryngol. 99, 369–378. 10.1177/000348949009900510 2337316

[26] Fredelius, L. , and Rask-Andersen, H. (1990). “ The role of macrophages in the disposal of degeneration products within the organ of Corti after acoustic overstimulation,” Acta Otolaryngol. 109, 76–82. 10.3109/00016489009107417 2309562

[27] Frye, M. D. , Yang, W. , Zhang, C. , Xiong, B. , and Hu, B. H. (2017). “ Dynamic activation of basilar membrane macrophages in response to chronic sensory cell degeneration in aging mouse cochleae,” Hear Res. 344, 125–134. 10.1016/j.heares.2016.11.003 27837652 PMC5239751

[28] Frye, M. D. , Zhang, C. , and Hu, B. H. (2018). “ Lower level noise exposure that produces only TTS modulates the immune homeostasis of cochlear macrophages,” J. Neuroimmunol. 323, 152–166. 10.1016/j.jneuroim.2018.06.019 30196827 PMC6132261

[29] Fujioka, M. , Kanzaki, S. , Okano, H. J. , Masuda, M. , Ogawa, K. , and Okano, H. (2006). “ Proinflammatory cytokines expression in noise-induced damaged cochlea,” J. Neurosci. Res. 83, 575–583. 10.1002/jnr.20764 16429448

[30] Fujioka, M. , Okamoto, Y. , Shinden, S. , Okano, H. J. , Okano, H. , Ogawa, K. , and Matsunaga, T. (2014a). “ Pharmacological inhibition of cochlear mitochondrial respiratory chain induces secondary inflammation in the lateral wall: A potential therapeutic target for sensorineural hearing loss,” PLoS One 9, e90089 10.1371/journal.pone.0090089 24614528 PMC3948682

[31] Fujioka, M. , Okano, H. , and Ogawa, K. (2014b). “ Inflammatory and immune responses in the cochlea: Potential therapeutic targets for sensorineural hearing loss,” Front. Pharmacol. 5, 287. 25566079 10.3389/fphar.2014.00287 PMC4274906

[32] Galli, S. J. , Borregaard, N. , and Wynn, T. A. (2011). “ Phenotypic and functional plasticity of cells of innate immunity: Macrophages, mast cells and neutrophils,” Nat. Immunol. 12, 1035–1044. 10.1038/ni.2109 22012443 PMC3412172

[33] Garcia-Berrocal, J. R. , Ibanez, A. , Rodriguez, A. , Gonzalez-Garcia, J. A. , Verdaguer, J. M. , Trinidad, A. , and Ramirez-Camacho, R. (2006). “ Alternatives to systemic steroid therapy for refractory immune-mediated inner ear disease: A physiopathologic approach,” Eur. Arch. Otorhinolaryngol. 263, 977–982. 10.1007/s00405-006-0096-9 16802138

[34] Gasteiger, G. , D'Osualdo, A. , Schubert, D. A. , Weber, A. , Bruscia, E. M. , and Hartl, D. (2017). “ Cellular innate immunity: An old game with new players,” J. Innate Immun. 9, 111–125. 10.1159/000453397 28006777 PMC6738785

[35] Gazquez, I. , Soto-Varela, A. , Aran, I. , Santos, S. , Batuecas, A. , Trinidad, G. , Perez-Garrigues, H. , Gonzalez-Oller, C. , Acosta, L. , and Lopez-Escamez, J. A. (2011). “ High prevalence of systemic autoimmune diseases in patients with Meniere's disease,” PLoS One 6, e26759 10.1371/journal.pone.0026759 22053211 PMC3203881

[36] Gloddek, B. , Bodmer, D. , Brors, D. , Keithley, E. M. , and Ryan, A. F. (2002). “ Induction of MHC class II antigens on cells of the inner ear,” Audiol. Neurootol. 7, 317–323. 10.1159/000066158 12463193

[37] Goodall, A. F. , and Siddiq, M. A. (2015). “ Current understanding of the pathogenesis of autoimmune inner ear disease: A review,” Clin. Otolaryngol. 40, 412–419. 10.1111/coa.12432 25847404

[38] Gratton, M. A. , Eleftheriadou, A. , Garcia, J. , Verduzco, E. , Martin, G. K. , Lonsbury-Martin, B. L. , and Vázquez, A. E. (2011). “ Noise-induced changes in gene expression in the cochleae of mice differing in their susceptibility to noise damage,” Hear Res. 277, 211–226. 10.1016/j.heares.2010.12.014 21187137 PMC3098916

[39] Grusby, M. J. , Johnson, R. S. , Papaioannou, V. E. , and Glimcher, L. H. (1991). “ Depletion of CD4+ T cells in major histocompatibility complex class II-deficient mice,” Science 253, 1417–1420. 10.1126/science.1910207 1910207

[40] Hakuba, N. , Hata, R. , Morizane, I. , Feng, G. , Shimizu, Y. , Fujita, K. , Yoshida, T. , Sakanaka, M. , and Gyo, K. (2005). “ Neural stem cells suppress the hearing threshold shift caused by cochlear ischemia,” Neuroreport 16, 1545–1549. 16148742

[41] Harris, J. P. (1984). “ Immunology of the inner ear: Evidence of local antibody production,” Ann. Otol. Rhinol. Laryngol. 93, 157–162. 10.1177/000348948409300211 6712089

[42] Harris, J. P. , and Ryan, A. F. (1984). “ Immunobiology of the inner ear,” Am. J. Otolaryngol. 5, 418–425. 10.1016/S0196-0709(84)80059-9 6400717

[43] Harris, J. P. , Weisman, M. H. , Derebery, J. M. , Espeland, M. A. , Gantz, B. J. , Gulya, A. J. , Hammerschlag, P. E. , Hannley, M. , Hughes, G. B. , Moscicki, R. , Nelson, R. A. , Niparko, J. K. , Rauch, S. D. , Telian, S. A. , and Brookhouser, P. E. (2003). “ Treatment of corticosteroid-responsive autoimmune inner ear disease with methotrexate: A randomized controlled trial,” JAMA 290, 1875–1883. 10.1001/jama.290.14.1875 14532316

[44] Hashimoto, S. , Billings, P. , Harris, J. P. , Firestein, G. S. , and Keithley, E. M. (2005). “ Innate immunity contributes to cochlear adaptive immune responses,” Audiol. Neurootol. 10, 35–43. 10.1159/000082306 15567913

[45] Hess, D. C. , Abe, T. , Hill, W. D. , Studdard, A. M. , Carothers, J. , Masuya, M. , Fleming, P. A. , Drake, C. J. , and Ogawa, M. (2004). “ Hematopoietic origin of microglial and perivascular cells in brain,” Exp. Neurol. 186, 134–144. 10.1016/j.expneurol.2003.11.005 15026252

[46] Hirose, K. , Discolo, C. M. , Keasler, J. R. , and Ransohoff, R. (2005). “ Mononuclear phagocytes migrate into the murine cochlea after acoustic trauma,” J. Comput. Neurol. 489, 180–194. 10.1002/cne.20619 15983998

[47] Hirose, K. , and Liberman, M. C. (2003). “ Lateral wall histopathology and endocochlear potential in the noise-damaged mouse cochlea,” J. Assoc. Res. Otolaryngol. 4, 339–352. 10.1007/s10162-002-3036-4 14690052 PMC1805786

[48] Hirose, K. , Rutherford, M. A. , and Warchol, M. E. (2017). “ Two cell populations participate in clearance of damaged hair cells from the sensory epithelia of the inner ear,” Hear Res. 352, 70–81. 10.1016/j.heares.2017.04.006 28526177 PMC5544544

[49] Hu, B. H. , Zhang, C. , and Frye, M. D. (2018). “ Immune cells and non-immune cells with immune function in mammalian cochleae,” Hear Res. 362, 14–24. 10.1016/j.heares.2017.12.009 29310977 PMC5911222

[50] Huang, Q. , Lin, B. , Liu, H. , Ma, X. , Mo, F. , Yu, W. , Li, L. , Li, H. , Tian, T. , Wu, D. , Shen, F. , Xing, J. , and Chen, Z. N. (2011). “ RNA-Seq analyses generate comprehensive transcriptomic landscape and reveal complex transcript patterns in hepatocellular carcinoma,” PLoS One 6, e26168 10.1371/journal.pone.0026168 22043308 PMC3197143

[51] Hughes, G. B. , Kinney, S. E. , Barna, B. P. , and Calabrese, L. H. (1983). “ Autoimmune reactivity in Meniere's disease: A preliminary report,” Laryngoscope 93, 410–417. 10.1002/lary.1983.93.4.410 6834965

[52] Hukee, M. J. , and Duvall, A. J. III. (1985). “ Cochlear vessel permeability to horseradish peroxidase in the normal and acoustically traumatized chinchilla: A reevaluation,” Ann. Otol. Rhinol. Laryngol. 94, 297–303. 4014952

[53] Hume, D. A. , Ross, I. L. , Himes, S. R. , Sasmono, R. T. , Wells, C. A. , and Ravasi, T. (2002). “ The mononuclear phagocyte system revisited,” J. Leukoc. Biol. 72, 621–627. 12377929

[54] Imai, Y. , Ibata, I. , Ito, D. , Ohsawa, K. , and Kohsaka, S. (1996). “ A novel gene iba1 in the major histocompatibility complex class III region encoding an EF hand protein expressed in a monocytic lineage,” Biochem. Biophys. Res. Commun. 224, 855–862. 10.1006/bbrc.1996.1112 8713135

[55] Iwai, H. , Lee, S. , Inaba, M. , Sugiura, K. , Baba, S. , Tomoda, K. , Yamashita, T. , and Ikehara, S. (2003). “ Correlation between accelerated presbycusis and decreased immune functions,” Exp. Gerontol. 38, 319–325. 10.1016/S0531-5565(02)00177-8 12581797

[56] Jabba, S. V. , Oelke, A. , Singh, R. , Maganti, R. J. , Fleming, S. , Wall, S. M. , Everett, L. A. , Green, E. D. , and Wangemann, P. (2006). “ Macrophage invasion contributes to degeneration of stria vascularis in Pendred syndrome mouse model,” BMC Med 4, 37 10.1186/1741-7015-4-37 17187680 PMC1796619

[57] Kalinec, G. M. , Lomberk, G. , Urrutia, R. A. , and Kalinec, F. (2017). “ Resolution of cochlear inflammation: Novel target for preventing or ameliorating drug-, noise- and age-related hearing loss,” Front. Cell Neurosci. 11, 192 10.3389/fncel.2017.00192 28736517 PMC5500902

[58] Kariko, K. , Ni, H. , Capodici, J. , Lamphier, M. , and Weissman, D. (2004). “ mRNA is an endogenous ligand for Toll-like receptor 3,” J. Biol. Chem. 279, 12542–12550. 10.1074/jbc.M310175200 14729660

[59] Kaur, T. , Zamani, D. , Tong, L. , Rubel, E. W. , Ohlemiller, K. K. , Hirose, K. , and Warchol, M. E. (2015). “ Fractalkine signaling regulates macrophage recruitment into the cochlea and promotes the survival of spiral ganglion neurons after selective hair cell lesion,” J. Neurosci. 35, 15050–15061. 10.1523/JNEUROSCI.2325-15.2015 26558776 PMC4642237

[60] Kesser, B. W. , and Lalwani, A. K. (2009). “ Gene therapy and stem cell transplantation: Strategies for hearing restoration,” Adv. Otorhinolaryngol. 66, 64–86. 10.1159/000218208 19494573

[61] Kirkegaard, M. , Murai, N. , Risling, M. , Suneson, A. , Jarlebark, L. , and Ulfendahl, M. (2006). “ Differential gene expression in the rat cochlea after exposure to impulse noise,” Neuroscience 142, 425–435. 10.1016/j.neuroscience.2006.06.037 16887274

[62] Kloss, C. U. , Werner, A. , Klein, M. A. , Shen, J. , Menuz, K. , Probst, J. C. , Kreutzberg, G. W. , and Raivich, G. (1999). “ Integrin family of cell adhesion molecules in the injured brain: Regulation and cellular localization in the normal and regenerating mouse facial motor nucleus,” J. Comput. Neurol. 411, 162–178. 10.1002/(SICI)1096-9861(19990816)411:1<162::AID-CNE12>3.0.CO;2-W 10404114

[63] Kreutzberg, G. W. (1996). “ Microglia: A sensor for pathological events in the CNS,” Trends Neurosci. 19, 312–318. 10.1016/0166-2236(96)10049-7 8843599

[64] Kujawa, S. G. , and Liberman, M. C. (2009). “ Adding insult to injury: Cochlear nerve degeneration after ‘temporary’ noise-induced hearing loss,” J. Neurosci. 29, 14077–14085. 10.1523/JNEUROSCI.2845-09.2009 19906956 PMC2812055

[65] Kumagai, Y. , and Akira, S. (2010). “ Identification and functions of pattern-recognition receptors,” J. Allergy Clin. Immunol. 125, 985–992. 10.1016/j.jaci.2010.01.058 20392481

[66] Kurabi, A. , Pak, K. , Ryan, A. F. , and Wasserman, S. I. (2016). “ Innate Immunity: Orchestrating Inflammation and Resolution of Otitis Media,” Curr. Allergy Asthma Reports 16, 6 10.1007/s11882-015-0585-2 PMC6752202 26732809

[67] Kuzmich, N. N. , Sivak, K. V. , Chubarev, V. N. , Porozov, Y. B. , Savateeva-Lyubimova, T. N. , and Peri, F. (2017). “ TLR4 signaling pathway modulators as potential therapeutics in inflammation and sepsis,” Vaccines 5, E34 10.3390/vaccines5040034 28976923 PMC5748601

[68] Lang, H. , Ebihara, Y. , Schmiedt, R. A. , Minamiguchi, H. , Zhou, D. , Smythe, N. , Liu, L. , Ogawa, M. , and Schulte, B. A. (2006). “ Contribution of bone marrow hematopoietic stem cells to adult mouse inner ear: Mesenchymal cells and fibrocytes,” J. Comput. Neurol. 496, 187–201. 10.1002/cne.20929 PMC2561311 16538683

[69] Lasak, J. M. , Sataloff, R. T. , Hawkshaw, M. , Carey, T. E. , Lyons, K. M. , and Spiegel, J. R. (2001). “ Autoimmune inner ear disease: Steroid and cytotoxic drug therapy,” Ear Nose Throat J. 80, 808–811, 815–806, 818 passim. 10.1177/014556130108001110 11816893

[70] Lawrence, T. , and Natoli, G. (2011). “ Transcriptional regulation of macrophage polarization: Enabling diversity with identity,” Nat. Rev. Immunol. 11, 750–761. 10.1038/nri3088 22025054

[71] Leichtle, A. , Hernandez, M. , Lee, J. , Pak, K. , Webster, N. J. , Wollenberg, B. , Wasserman, S. I. , and Ryan, A. F. (2012). “ The role of DNA sensing and innate immune receptor TLR9 in otitis media,” Innate Immun. 18, 3–13. 10.1177/1753425910393539 21239460 PMC4041324

[72] Liberman, M. C. , and Mulroy, M. J. (1982). “ Acute and chronic effects of acoustic trauma: Cochlear pathology and auditory nerve pathophysiology pathophysiology,” in *New Perspectives on Noise-Induced Hearing Loss*, edited by Hamernik R. P., Henderson D., and Salvi R. ( Raven, New York), pp. 105–136.

[73] Liu, H. , Pecka, J. L. , Zhang, Q. , Soukup, G. A. , Beisel, K. W. , and He, D. Z. (2014). “ Characterization of transcriptomes of cochlear inner and outer hair cells,” J. Neurosci. 34, 11085–11095. 10.1523/JNEUROSCI.1690-14.2014 25122905 PMC4131018

[74] Liu, Y. C. , Rubin, R. , and Sataloff, R. T. (2011). “ Treatment-refractory autoimmune sensorineural hearing loss: Response to infliximab,” Ear Nose Throat J. 90, 23–28. 10.1177/014556131109000107 21229506

[75] Mark, A. S. , Seltzer, S. , Nelson-Drake, J. , Chapman, J. C. , Fitzgerald, D. C. , and Gulya, A. J. (1992). “ Labyrinthine enhancement on gadolinium-enhanced magnetic resonance imaging in sudden deafness and vertigo: Correlation with audiologic and electronystagmographic studies,” Ann. Otol. Rhinol. Laryngol. 101, 459–464. 10.1177/000348949210100601 1610062

[76] Matern, M. , Vijayakumar, S. , Margulies, Z. , Milon, B. , Song, Y. , Elkon, R. , Zhang, X. , Jones, S. M. , and Hertzano, R. (2017). “ Gfi1(Cre) mice have early onset progressive hearing loss and induce recombination in numerous inner ear non-hair cells,” Sci. Rep. 7, 42079 10.1038/srep42079 28181545 PMC5299610

[77] Matsubara, A. , Laake, J. H. , Davanger, S. , Usami, S. , and Ottersen, O. P. (1996). “ Organization of AMPA receptor subunits at a glutamate synapse: A quantitative immunogold analysis of hair cell synapses in the rat organ of Corti,” J. Neurosci. 16, 4457–4467. 10.1523/JNEUROSCI.16-14-04457.1996 8699256 PMC6578857

[78] Matsunaga, T. , Kanzaki, J. , Masuda, M. , and Hosoda, Y. (1995). “ Ultrastructure of the vestibular dark cell area in patients with acoustic neurinoma,” ORL J. Otorhinolaryngol. Relat. Spec. 57, 182–188. 10.1159/000276736 7478450

[79] Matteson, E. L. , Choi, H. K. , Poe, D. S. , Wise, C. , Lowe, V. J. , McDonald, T. J. , and Rahman, M. U. (2005). “ Etanercept therapy for immune-mediated cochleovestibular disorders: A multi-center, open-label, pilot study,” Arthritis Rheum. 53, 337–342. 10.1002/art.21179 15934127

[80] Matteson, E. L. , Fabry, D. A. , Facer, G. W. , Beatty, C. W. , Driscoll, C. L. , Strome, S. E. , and McDonald, T. J. (2001). “ Open trial of methotrexate as treatment for autoimmune hearing loss,” Arthritis Rheum. 45, 146–150. 10.1002/1529-0131(200104)45:2<146::AID-ANR167>3.0.CO;2-I 11324778

[81] McCabe, B. F. (1989). “ Autoimmune inner ear disease: Therapy,” Am. J. Otol. 10, 196–197. 2750868

[82] Mistry, P. , Laird, M. H. , Schwarz, R. S. , Greene, S. , Dyson, T. , Snyder, G. A. , Xiao, T. S. , Chauhan, J. , Fletcher, S. , Toshchakov, V. Y. , MacKerell, A. D., Jr. , and Vogel, S. N. (2015). “ Inhibition of TLR2 signaling by small molecule inhibitors targeting a pocket within the TLR2 TIR domain,” Proc. Natl. Acad. Sci. U.S.A. 112, 5455–5460. 10.1073/pnas.1422576112 25870276 PMC4418912

[83] Miyao, M. , Firestein, G. S. , and Keithley, E. M. (2008). “ Acoustic trauma augments the cochlear immune response to antigen,” Laryngoscope 118, 1801–1808. 10.1097/MLG.0b013e31817e2c27 18806477 PMC2832795

[84] Mogensen, T. H. (2009). “ Pathogen recognition and inflammatory signaling in innate immune defenses,” Clin. Microbiol. Rev. 22, 240–273. 10.1128/CMR.00046-08 19366914 PMC2668232

[85] Mohler, K. M. , Torrance, D. S. , Smith, C. A. , Goodwin, R. G. , Stremler, K. E. , Fung, V. P. , Madani, H. , and Widmer, M. B. (1993). “ Soluble tumor necrosis factor (TNF) receptors are effective therapeutic agents in lethal endotoxemia and function simultaneously as both TNF carriers and TNF antagonists,” J. Immunol. 151, 1548–1561. 8393046

[86] Morovic Vergles, J. , Radic, M. , Kovacic, J. , and Salamon, L. (2010). “ Successful use of adalimumab for treating rheumatoid arthritis with autoimmune sensorineural hearing loss: Two birds with one stone,” J. Rheumatol. 37, 1080–1081. 10.3899/jrheum.091342 20439536

[87] Moscicki, R. A. , San Martin, J. E. , Quintero, C. H. , Rauch, S. D. , Nadol, J. B., Jr. , and Bloch, K. J. (1994). “ Serum antibody to inner ear proteins in patients with progressive hearing loss. Correlation with disease activity and response to corticosteroid treatment,” JAMA 272, 611–616. 10.1001/jama.1994.03520080053043 8057517

[88] Murray, P. J. , and Wynn, T. A. (2011). “ Obstacles and opportunities for understanding macrophage polarization,” J. Leukoc. Biol. 89, 557–563. 10.1189/jlb.0710409 21248152 PMC3058818

[89] Nimmerjahn, A. , Kirchhoff, F. , and Helmchen, F. (2005). “ Resting microglial cells are highly dynamic surveillants of brain parenchyma in vivo,” Science 308, 1314–1318. 10.1126/science.1110647 15831717

[90] Nishimoto, N. , and Kishimoto, T. (2006). “ Interleukin 6: From bench to bedside,” Nat. Clin. Pract. Rheumatol. 2, 619–626. 10.1038/ncprheum0338 17075601

[91] Oh, S. , Woo, J. I. , Lim, D. J. , and Moon, S. K. (2012). “ ERK2-dependent activation of c-Jun is required for nontypeable Haemophilus influenzae-induced CXCL2 upregulation in inner ear fibrocytes,” J. Immunol. 188, 3496–3505. 10.4049/jimmunol.1103182 22379036 PMC3311727

[92] Okano, T. , Nakagawa, T. , Kita, T. , Endo, T. , and Ito, J. (2006). “ Cell-gene delivery of brain-derived neurotrophic factor to the mouse inner ear,” Mol. Ther. 14, 866–871. 10.1016/j.ymthe.2006.06.012 16956795

[93] Okano, T. , Nakagawa, T. , Kita, T. , Kada, S. , Yoshimoto, M. , Nakahata, T. , and Ito, J. (2008). “ Bone marrow-derived cells expressing Iba1 are constitutively present as resident tissue macrophages in the mouse cochlea,” J. Neurosci. Res. 86, 1758–1767. 10.1002/jnr.21625 18253944

[94] Patel, M. , Hu, Z. , Bard, J. , Jamison, J. , Cai, Q. , and Hu, B. H. (2013). “ Transcriptome characterization by RNA-Seq reveals the involvement of the complement components in noise-traumatized rat cochleae,” Neuroscience 248, 1–16. 10.1016/j.neuroscience.2013.05.038 23727008 PMC3868636

[95] Perera, A. P. , Fernando, R. , Shinde, T. , Gundamaraju, R. , Southam, B. , Sohal, S. S. , Robertson, A. A. B. , Schroder, K. , Kunde, D. , and Eri, R. (2018). “ MCC950, a specific small molecule inhibitor of NLRP3 inflammasome attenuates colonic inflammation in spontaneous colitis mice,” Sci. Rep. 8, 8618 10.1038/s41598-018-26775-w 29872077 PMC5988655

[96] Peters, A. , and Swan, R. C. (1979). “ The choroid plexus of the mature and aging rat: The choroidal epithelium,” Anat. Rec. 194, 325–353. 10.1002/ar.1091940303 475003

[97] Psillas, G. , Pavlidis, P. , Karvelis, I. , Kekes, G. , Vital, V. , and Constantinidis, J. (2008). “ Potential efficacy of early treatment of acute acoustic trauma with steroids and piracetam after gunshot noise,” Eur. Arch. Otorhinolaryngol. 265, 1465–1469. 10.1007/s00405-008-0689-6 18463885

[98] Rahman, M. U. , Poe, D. S. , and Choi, H. K. (2001). “ Etanercept therapy for immune-mediated cochleovestibular disorders: Preliminary results in a pilot study,” Otol. Neurotol. 22, 619–624. 10.1097/00129492-200109000-00010 11568668

[99] Raivich, G. , Bohatschek, M. , Kloss, C. U. , Werner, A. , Jones, L. L. , and Kreutzberg, G. W. (1999). “ Neuroglial activation repertoire in the injured brain: Graded response, molecular mechanisms and cues to physiological function,” Brain Res. Brain Res. Rev. 30, 77–105. 10.1016/S0165-0173(99)00007-7 10407127

[100] Ramprasad, M. P. , Terpstra, V. , Kondratenko, N. , Quehenberger, O. , and Steinberg, D. (1996). “ Cell surface expression of mouse macrosialin and human CD68 and their role as macrophage receptors for oxidized low density lipoprotein,” Proc. Natl. Acad. Sci. U.S.A. 93, 14833–14838. 10.1073/pnas.93.25.14833 8962141 PMC26222

[101] Rask-Andersen, H. , and Stahle, J. (1979). “ Lymphocyte-macrophage activity in the endolymphatic sac. An ultrastructural study of the rugose endolymphatic sac in the guinea pig,” ORL J. Otorhinolaryngol. Relat. Spec. 41, 177–192. 10.1159/000275458 574240

[102] Robbin, R. P. , and Thompson, M. H. (1978). “ Effects of putative transmitters on afferent cochlear transmission,” Ann. Otol. Rhinol. Laryngol. 87, 185–190. 10.1177/000348947808700207 206175

[103] Robertson, D. (1983). “ Functional significance of dendritic swelling after loud sounds in the guinea pig cochlea,” Hear Res. 9, 263–278. 10.1016/0378-5955(83)90031-X 6841283

[104] Roers, A. , Hiller, B. , and Hornung, V. (2016). “ Recognition of endogenous nucleic acids by the innate immune system,” Immunity 44, 739–754. 10.1016/j.immuni.2016.04.002 27096317

[105] Salley, L. H., Jr. , Grimm, M. , Sismanis, A. , Spencer, R. F. , and Wise, C. M. (2001). “ Methotrexate in the management of immune mediated cochleovesitibular disorders: Clinical experience with 53 patients,” J. Rheumatol. 28, 1037–1040. 11361185

[106] Sato, E. , Shick, H. E. , Ransohoff, R. M. , and Hirose, K. (2008). “ Repopulation of cochlear macrophages in murine hematopoietic progenitor cell chimeras: The role of CX3CR1,” J. Comput. Neurol. 506, 930–942. 10.1002/cne.21583 18085589

[107] Satoh, H. , Firestein, G. S. , Billings, P. B. , Harris, J. P. , and Keithley, E. M. (2002). “ Tumor necrosis factor-alpha, an initiator, and etanercept, an inhibitor of cochlear inflammation,” Laryngoscope 112, 1627–1634. 10.1097/00005537-200209000-00019 12352677

[108] Satoh, H. , Firestein, G. S. , Billings, P. B. , Harris, J. P. , and Keithley, E. M. (2003). “ Proinflammatory cytokine expression in the endolymphatic sac during inner ear inflammation,” J. Assoc. Res. Otolaryngol. 4, 139–147. 10.1007/s10162-002-3025-7 12943369 PMC3202716

[109] Sautter, N. , Shick, E. , Ransohoff, R. , Charo, I. , and Hirose, K. (2006). “ CC chemokine receptor 2 is protective against noise-induced hair cell death: Studies in CX3CR1+/GFP mice,” J. Assoc. Res. Otolaryngol. 7, 361–372. 10.1007/s10162-006-0051-x 17075702 PMC2504633

[110] Schaefer, L. (2014). “ Complexity of danger: The diverse nature of damage-associated molecular patterns,” J. Biol. Chem. 289, 35237–35245. 10.1074/jbc.R114.619304 25391648 PMC4271212

[111] Shi, X. (2009). “ Cochlear pericyte responses to acoustic trauma and the involvement of hypoxia-inducible factor-1alpha and vascular endothelial growth factor,” Am. J. Pathol. 174, 1692–1704. 10.2353/ajpath.2009.080739 19349367 PMC2671258

[112] Shi, X. (2010). “ Resident macrophages in the cochlear blood-labyrinth barrier and their renewal via migration of bone-marrow-derived cells,” Cell Tissue Res. 342, 21–30. 10.1007/s00441-010-1040-2 20838812

[113] Siddiqui, M. A. , and Scott, L. J. (2005). “ Infliximab: A review of its use in Crohn's disease and rheumatoid arthritis,” Drugs 65, 2179–2208. 10.2165/00003495-200565150-00014 16225377

[114] Smith, M. J. , and Koch, G. L. (1987). “ Differential expression of murine macrophage surface glycoprotein antigens in intracellular membranes,” J. Cell Sci. 87(1), 113–119. 3312248 10.1242/jcs.87.1.113

[115] Spear, S. A. , and Schwartz, S. R. (2011). “ Intratympanic steroids for sudden sensorineural hearing loss,” Otolaryngol. Head Neck Surg. 145, 534–543. 10.1177/0194599811419466 21873598

[116] Spoendlin, H. (1971). “ Primary structural changes in the organ of Corti after acoustic overstimulation,” Acta Otolaryngol. 71, 166–176. 10.3109/00016487109125346 5577011

[117] Steinman, R. M. (1991). “ The dendritic cell system and its role in immunogenicity,” Ann. Rev. Immunol. 9, 271–296. 10.1146/annurev.iy.09.040191.001415 1910679

[118] Stence, N. , Waite, M. , and Dailey, M. E. (2001). “ Dynamics of microglial activation: A confocal time-lapse analysis in hippocampal slices,” Glia 33, 256–266. 10.1002/1098-1136(200103)33:3<256::AID-GLIA1024>3.0.CO;2-J 11241743

[119] Stokroos, R. J. , Albers, F. W. , Krikke, A. P. , and Casselman, J. W. (1998). “ Magnetic resonance imaging of the inner ear in patients with idiopathic sudden sensorineural hearing loss,” Eur. Arch. Otorhinolaryngol. 255, 433–436. 10.1007/s004050050093 9833208

[120] Sulkowski, W. , Starzynski, Z. , and Szeszenia-Dabrowska, N. (1981). “ Epidemiology of occupational noise-induced hearing loss in Poland throughout 1971–1979,” Med. Pr. 32, 9–16. 7289864

[121] Swain, S. L. (1983). “ T cell subsets and the recognition of MHC class,” Immunol. Rev. 74, 129–142. 10.1111/j.1600-065X.1983.tb01087.x 6226585

[122] Takahashi, M. , and Harris, J. P. (1988). “ Analysis of immunocompetent cells following inner ear immunostimulation,” Laryngoscope 98, 1133–1138. 10.1288/00005537-198810000-00018 2971844

[123] Takahashi, K. , Kusakari, J. , Kimura, S. , Wada, T. , and Hara, A. (1996). “ The effect of methylprednisolone on acoustic trauma,” Acta Otolaryngol. 116, 209–212. 10.3109/00016489609137825 8725516

[124] Takemura, N. , Kawasaki, T. , Kunisawa, J. , Sato, S. , Lamichhane, A. , Kobiyama, K. , Aoshi, T. , Ito, J. , Mizuguchi, K. , Karuppuchamy, T. , Matsunaga, K. , Miyatake, S. , Mori, N. , Tsujimura, T. , Satoh, T. , Kumagai, Y. , Kawai, T. , Standley, D. M. , Ishii, K. J. , Kiyono, H. , Akira, S. , and Uematsu, S. (2014). “ Blockade of TLR3 protects mice from lethal radiation-induced gastrointestinal syndrome,” Nat. Commun. 5, 3492 10.1038/ncomms4492 24637670 PMC3959210

[125] Takeuchi, O. , and Akira, S. (2010). “ Pattern recognition receptors and inflammation,” Cell 140, 805–820. 10.1016/j.cell.2010.01.022 20303872

[126] Tan, W. J. , Thorne, P. R. , and Vlajkovic, S. M. (2016). “ Characterisation of cochlear inflammation in mice following acute and chronic noise exposure,” Histochem. Cell Biol. 146, 219–230. 10.1007/s00418-016-1436-5 27109494

[127] Tang, D. , Kang, R. , Coyne, C. B. , Zeh, H. J. , and Lotze, M. T. (2012). “ PAMPs and DAMPs: Signal 0s that spur autophagy and immunity,” Immunol. Rev. 249, 158–175. 10.1111/j.1600-065X.2012.01146.x 22889221 PMC3662247

[128] Taylor, W. , Pearson, J. , Mair, A. , and Burns, W. (1965). “ Study of noise and hearing in jute weaving,” J. Acoust. Soc. Am. 38, 113–120. 10.1121/1.1909580 14347600

[129] Tornabene, S. V. , Sato, K. , Pham, L. , Billings, P. , and Keithley, E. M. (2006). “ Immune cell recruitment following acoustic trauma,” Hear Res. 222, 115–124. 10.1016/j.heares.2006.09.004 17081714

[130] Toubi, E. , Ben-David, J. , Kessel, A. , Halas, K. , Sabo, E. , and Luntz, M. (2004). “ Immune-mediated disorders associated with idiopathic sudden sensorineural hearing loss,” Ann. Otol. Rhinol. Laryngol. 113, 445–449. 10.1177/000348940411300605 15224826

[131] Tran, H. T. , Liu, Y. , Zurita, A. J. , Lin, Y. , Baker-Neblett, K. L. , Martin, A. M. , Figlin, R. A. , Hutson, T. E. , Sternberg, C. N. , Amado, R. G. , Pandite, L. N. , and Heymach, J. V. (2012). “ Prognostic or predictive plasma cytokines and angiogenic factors for patients treated with pazopanib for metastatic renal-cell cancer: A retrospective analysis of phase 2 and phase 3 trials,” The Lancet: Oncol. 13, 827–837. 10.1016/S1470-2045(12)70241-3 22759480

[132] Unanue, E. R. (1984). “ Antigen-presenting function of the macrophage,” Ann. Rev. Immunol. 2, 395–428. 10.1146/annurev.iy.02.040184.002143 6242349

[133] Vannella, K. M. , and Wynn, T. A. (2017). “ Mechanisms of organ injury and repair by macrophages,” Ann. Rev. Physiol. 79, 593–617. 10.1146/annurev-physiol-022516-034356 27959618

[134] Van Wijk, F. , Staecker, H. , Keithley, E. , and Lefebvre, P. P. (2006). “ Local perfusion of the tumor necrosis factor alpha blocker infliximab to the inner ear improves autoimmune neurosensory hearing loss,” Audiol. Neurootol. 11, 357–365. 10.1159/000095897 16988499

[135] Varol, C. , Mildner, A. , and Jung, S. (2015). “ Macrophages: Development and tissue specialization,” Ann. Rev. Immunol. 33, 643–675. 10.1146/annurev-immunol-032414-112220 25861979

[136] Vaughan, D. W. , and Peters, A. (1974). “ Neuroglial cells in the cerebral cortex of rats from young adulthood to old age: An electron microscope study,” J. Neurocytol. 3, 405–429. 10.1007/BF01098730 4373545

[137] Vethanayagam, R. R. , Yang, W. , Dong, Y. , and Hu, B. H. (2016). “ Toll-like receptor 4 modulates the cochlear immune response to acoustic injury,” Cell Death Dis. 7, e2245 10.1038/cddis.2016.156 27253409 PMC5143385

[138] Wakabayashi, K. , Fujioka, M. , Kanzaki, S. , Okano, H. J. , Shibata, S. , Yamashita, D. , Masuda, M. , Mihara, M. , Ohsugi, Y. , Ogawa, K. , and Okano, H. (2010). “ Blockade of interleukin-6 signaling suppressed cochlear inflammatory response and improved hearing impairment in noise-damaged mice cochlea,” Neurosci. Res. 66, 345–352. 10.1016/j.neures.2009.12.008 20026135

[139] Wang, Y. , Hirose, K. , and Liberman, M. C. (2002). “ Dynamics of noise-induced cellular injury and repair in the mouse cochlea,” J. Assoc. Res. Otolaryngol. 3, 248–268. 10.1007/s101620020028 12382101 PMC3202415

[140] Willrich, M. A. , Murray, D. L. , and Snyder, M. R. (2015). “ Tumor necrosis factor inhibitors: Clinical utility in autoimmune diseases,” Trans. Res. 165, 270–282. 10.1016/j.trsl.2014.09.006 25305470

[141] Wood, M. B. , and Zuo, J. (2017). “ The contribution of immune infiltrates to ototoxicity and cochlear hair cell loss,” Front. Cell Neurosci. 11, 106 10.3389/fncel.2017.00106 28446866 PMC5388681

[142] Yang, S. , Cai, Q. , Vethanayagam, R. R. , Wang, J. , Yang, W. , and Hu, B. H. (2016). “ Immune defense is the primary function associated with the differentially expressed genes in the cochlea following acoustic trauma,” Hear Res. 333, 283–294. 10.1016/j.heares.2015.10.010 26520584 PMC4798880

[143] Yang, W. , Vethanayagam, R. R. , Dong, Y. , Cai, Q. , and Hu, B. H. (2015). “ Activation of the antigen presentation function of mononuclear phagocyte populations associated with the basilar membrane of the cochlea after acoustic overstimulation,” Neuroscience 303, 1–15. 10.1016/j.neuroscience.2015.05.081 26102003 PMC4532582

[144] Yimtae, K. , Song, H. , Billings, P. , Harris, J. P. , and Keithley, E. M. (2001). “ Connection between the inner ear and the lymphatic system,” Laryngoscope 111, 1631–1635. 10.1097/00005537-200109000-00026 11568618

[145] Yin, J. , Valin, K. L. , Dixon, M. L. , and Leavenworth, J. W. (2017). “ The role of microglia and macrophages in CNS homeostasis, autoimmunity, and cancer,” J. Immunol. Res. 2017, 5150678 10.1155/2017/5150678 29410971 PMC5749282

[146] Young, R. W. , and Bok, D. (1969). “ Participation of the retinal pigment epithelium in the rod outer segment renewal process,” J. Cell Biol. 42, 392–403. 10.1083/jcb.42.2.392 5792328 PMC2107669

[147] Zeitoun, H. , Beckman, J. G. , Arts, H. A. , Lansford, C. D. , Lee, D. S. , El-Kashlan, H. K. , Telian, S. A. , Denny, D. E. , Ramakrishnan, A. , Nair, T. S. , Disher, M. J. , Sataloff, R. T. , Fisher, S. G. , and Carey, T. E. (2005). “ Corticosteroid response and supporting cell antibody in autoimmune hearing loss,” Arch. Otolaryngol. Head Neck Surg. 131, 665–672. 10.1001/archotol.131.8.665 16103296

[148] Zhang, W. , Dai, M. , Fridberger, A. , Hassan, A. , Degagne, J. , Neng, L. , Zhang, F. , He, W. , Ren, T. , Trune, D. , Auer, M. , and Shi, X. (2012). “ Perivascular-resident macrophage-like melanocytes in the inner ear are essential for the integrity of the intrastrial fluid-blood barrier,” Proc. Natl. Acad. Sci. U.S.A. 109, 10388–10393. 10.1073/pnas.1205210109 22689949 PMC3387119

[149] Zhang, C. , Sun, W. , Li, J. , Xiong, B. , Frye, M. D. , Ding, D. , Salvi, R. , Kim, M.-J. , Someya, S. , and Hu, B. H. (2017). “ Loss of sestrin 2 potentiates the early onset of age-related sensory cell degeneration in the cochlea,” Neuroscience 361, 179–191. 10.1016/j.neuroscience.2017.08.015 28818524 PMC5605466

[150] Zhao, L. , Lee, J. Y. , and Hwang, D. H. (2011). “ Inhibition of pattern recognition receptor-mediated inflammation by bioactive phytochemicals,” Nutr. Rev. 69, 310–320. 10.1111/j.1753-4887.2011.00394.x 21631512 PMC3881972

[151] Zhou, Y. , Zheng, G. , Zheng, H. , Zhou, R. , Zhu, X. , and Zhang, Q. (2013). “ Primary observation of early transtympanic steroid injection in patients with delayed treatment of noise-induced hearing loss,” Audiol. Neurootol. 18, 89–94. 10.1159/000345208 23208457

